# One-and-a-half nostril endoscopic transsphenoidal approach for pituitary adenomas—a technical report

**DOI:** 10.1186/s40463-016-0174-y

**Published:** 2016-11-15

**Authors:** Guodao Wen, Chao Tang, Chunyu Zhong, Junyang Li, Zixiang Cong, Yuan Zhou, Kaidong Liu, Yong Zhang, Mamatemin Tohti, Chiyuan Ma

**Affiliations:** 1 Department of Neurosurgery, Jinling Hospital, School of Medicine, Nanjing University, 305 East Zhongshan Road, Nanjing, 210002 Jiangsu Province People’s Republic of China; 2 School of Medicine, Nanjing medical University, 104 Hanzhong Road, Nanjing, 210002 People’s Republic of China; 3 Department of ear-nose-throat, Jinling Hospital, School of Medicine, Nanjing University, 305 East Zhongshan Road, Nanjing, 210002 People’s Republic of China; 4 Department of Neurosurgery, The People’s Hospital of Xinjiang Uygur Autonomous Region, 91 Tianchi Road, Urumqi, 830001 People’s Republic of China

**Keywords:** One-and-a-half nostril, Endonasal endoscopic transsphenoidal approach, The “rescue” nasoseptal flap, Trauma of nose, Manipulation space

## Abstract

**Background:**

Binostril endoscopic transsphenoidal approach (BETA) provides sufficient manipulation space and wide endoscopic vision, although it increases the trauma of nose. Mononostril endoscopic transsphenoidal approach (META) has minimal trauma of nose, at the expense of space within the operation. We describe a one-and-a-half nostril endoscopic transsphenoidal approach (OETA) that combines the advantages of BETA and META.

**Methods:**

We introduced OETA for pituitary adenomas with a detailed technical description. A retrospective analysis was also performed on 57 consecutive patients who underwent one-and-a-half nostril endoscopic transsphenoidal surgery between March 2014 and June 2015 at Jinling hospital.

**Results:**

The gross total resection rate was 79%. The gross complete resection rate of Knosp grade 3 tumors were 63.6, and 27.3% in grade 4 tumors. Postoperative hormone remission was achieved in 14 out of 18 (77.8%) patients with secreting adenomas. Postoperative abnormal visual function improvement was achieved in 23 out of 32 patients (73%) with preoperative visual dysfunction. The overall intra-operative CSF leak was 17.5%, with the postoperative CSF leak decreased to 3.5% after the sellar reconstruction with the unilateral “rescue” nasoseptal flap procedure. The main sinonasal complaints 2 weeks after surgery were: loss of sense of smell (28%), decrease in sense of taste (4%), trouble breathing during the day (18%), thick nasal discharge (36%), post nasal discharge (8%), dried nasal material (6%), and headache (6%). Three months after surgery, there were no reports of decrease of taste, post nasal discharge, or dried nasal material. Other complaints were decreased significantly. Six months after surgery, the main complaints of sinonasal quality of life were negligible, and overall health status was near complete recovery to preoperative status.

**Conclusions:**

The one-and-a-half nostril endoscopic transsphenoidal approach for pituitary adenomas is a simple and reliable technique. It provides not only a sufficient surgical corridor for a 2-surgeon/4 or 3-hands technique, but also ensures minimal invasion of the nasal canal.

## Background

Over the past several decades, the endonasalendoscopic transsphenoidal approach (ETSS) has gradually become the preferred surgical option for most pituitary adenomas [1, 2]. This approach offers improved illumination and superior panoramic visualization of the sella and the surrounding structures [3–5]. Compared with traditional sublabial or transseptal microscopic approach, ETSS offers minimal invasiveness, fewer complications, and overall improved outcomes [6, 7]. Traditionally, there are two approaches to endoscopic transsphenoidal surgery for pituitary adenomas: the two nostrils (binostril) approach and the one nostril (mononostril) approach [8, 9]. Both of the approaches have positives and negatives associated with them.

The binostril approach with a 2-surgeon/4- or 3-hands technique can provide a sufficient manipulation space and a wider endoscopic vision, which helps to avoid interference between instruments [10]. To create the surgical corridor successfully, the surgeon dissects both nasal cavities and resects parts of the posterior nasal septum. Obviously, there are increased risks of rhinological complications and postoperative discomfort. Recent studies have shown that BETA presents poor early-postoperative sinonasal QOL and a significant olfactory alteration [6, 11, 12]. Many cases reported better results using the mononostril approach [13, 14]. This technique spares one nostril from dissection, thus avoiding large resection and tissue manipulation. However, in the mononostril approach, surgical freedom is more restricted by the crowded nasal corridor and there are conflicts between the endoscope and dissecting instruments. Moreover, the presence of the nasal septum medially and the prominences of the nasal turbinates prevent surgical instruments from being angled toward the ipsilateral parasellar region [15].

In this technical report, we describe a one-and-a-half nostril endoscopic transsphenoidal approach (OETA) that combines the advantages of BETA and META. This approach maintains sufficient surgical freedom and retains the benefits of a 2-surgeon/4- or 3-hands technique, while preserving as much nasal septal mucosa as possible. Likewise, it possesses minimal nasal invasiveness similar to the mononstril approach.

## Methods

We retrospectively reviewed all patients that underwent the one-and-a-half nostril endoscopic transsphenoidal pituitary adenoma surgery at Jinling hospital, performed by a senior neurosurgeon (CY M) from March 2014 to June 2015. The medical records were reviewed for patient demographics, tumor characteristics, surgical outcomes, complications, and postoperative follow-up. The surgical technique of the OETA is described below.

### Surgical technique

The patient’s head was angled 20°–30° towards the left shoulder, using a horseshoe head-holder, while under general anesthesia and endotracheal intubation. The surgeon worked on the right side of the patient and routinely used the right nasal cavity as a pathway for a 30° endoscope (Karl Storz, Tuttlingen, Germany). The bilateral nasal cavities were packed with cottonoids containing 1% lidocaine for several minutes. No nasal speculum was used. The right inferior and middle turbinates were out-fractured bilaterally, which enabled a sufficiently large approach to the sphenoethmoidal recess. After advancing an endoscope over the right middle turbinate, the sphenoidostium was observed at posterosuperior medial to superior turbinate (Fig. 3a).

**Fig. 1:**
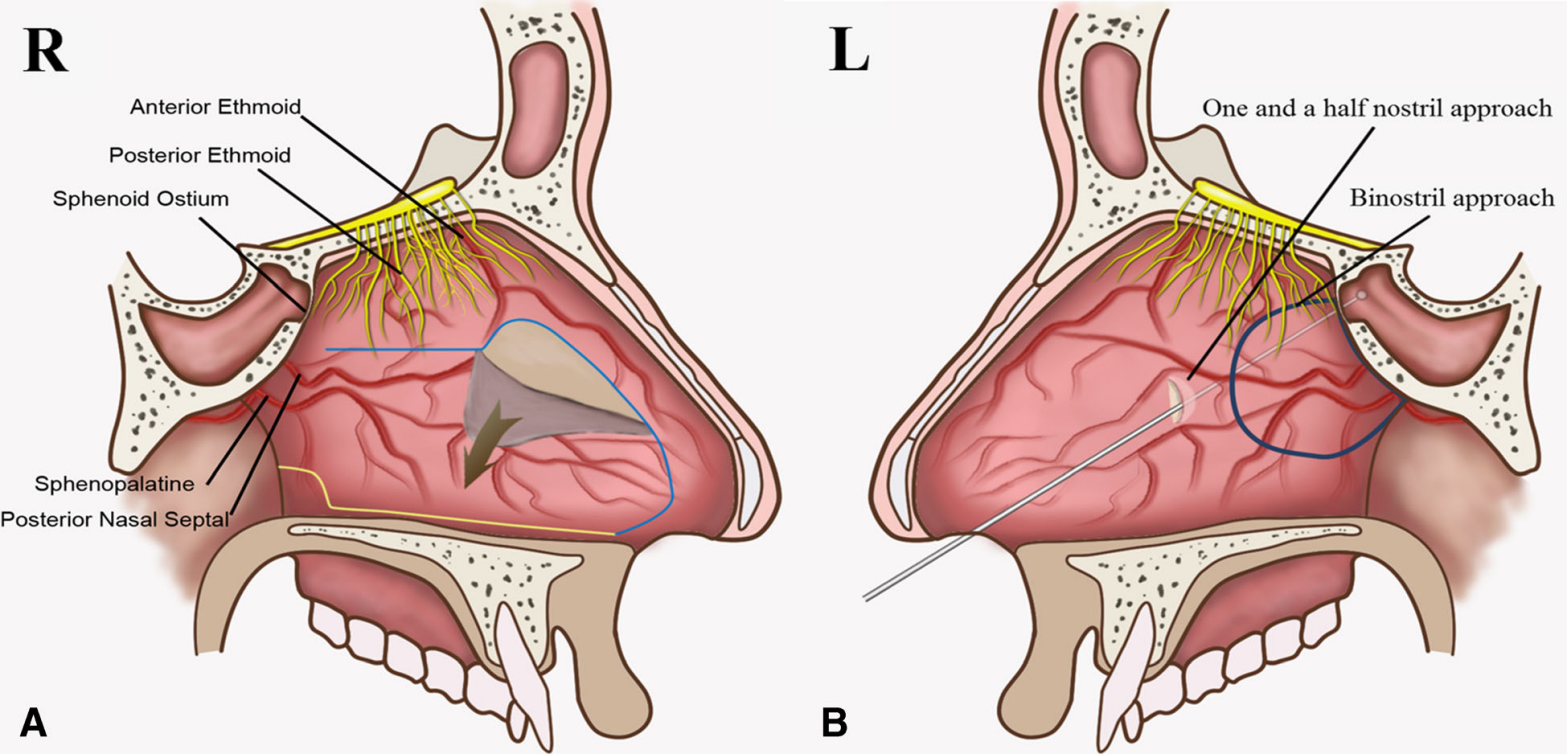
Illustration of the one-and-a-half nostril endoscopic transsphenoidal approach. **a** Creating the right “rescue” nasoseptal flap. The *blue line* shows mucosal cutting line: from 2 to 3 mm below the sphenoid ostium to the intercutaneomucous point of the nasal vestibule. If anasoseptal flap is necessary, the yellow mucosal cutting line is made: along the floor of the nasal cavity from the choanae to the front of the first incision. **b** One-and-a-half approach: a vertical incision of the left nasal septal mucosa was made, which extended approximately 2 cm at the anterior-level of the middle turbinate. Binostril approach: a part of left posterior septal mucosa was necessarily resected

Then, the right “rescue” nasoseptal flap was created with unipolar electrocautery (Fig. 1a). An anterior vertical incision was performed along the perpendicular plate of the ethmoid and posterior nasal septum, from 2 to 3 mm below the sphenoid ostium to the intercutaneomucous point of the nasal vestibule. To preserve the olfactory epithelium, the superior incision to the sphenoid ostium must be 1 to 2 cm below the most superior aspect of the septum (Fig. 3b–d). The unilateral “rescue” nasoseptal flap was gently reflected forward submucosally and folded into the floor of nasal cavity outside of the operative field of vision (Fig. 3e). If this nasoseptal flap was necessary for sellar floor reconstruction at the time of closure, a second incision was made along the floor of the nasal cavity from the choanae to the front of the first incision.

The next operation was to resect the bony nasal septum using a dissector. The vomer was fractured across at the base, and typically removed in one piece so as to preserve the bone for sellar floor reconstruction. The resecting was continued posteriorly until the sphenoid rostrum was visible. The left nasal septum mucosa was then pushed towards left cavity to make the left sphenoid ostium visible.

**Fig. 2:**
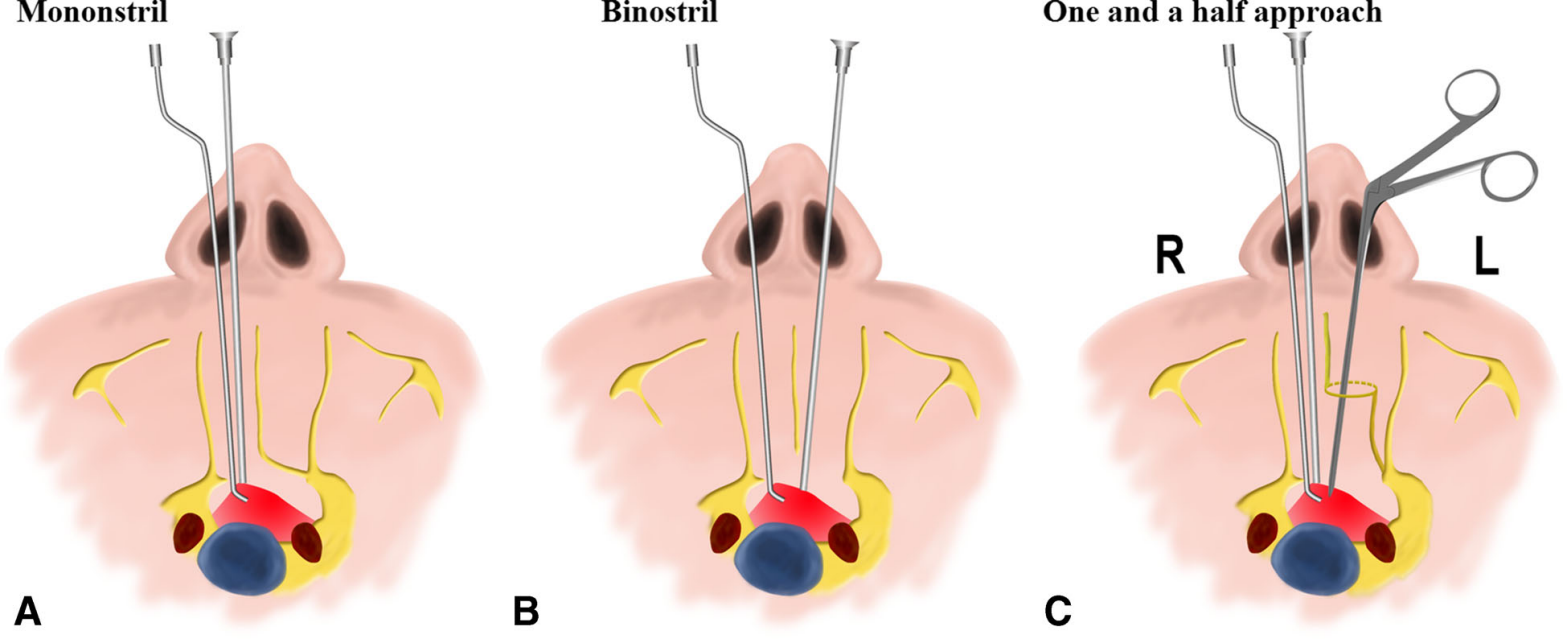
The difference between mononostril, one-and-a-half nostril and binostril approach. **a** Mononostril: a part of unilateral posterior septal mucosa was need incision and the contralateral mucosa was integral maintained; all surgical instruments manipulation only in a nasal cavitie. **b** One-and-a-half approach:the unilateral septal mucosa was required incising for a “rescue” nasoseptal flap, and the contralateral mucosa just needed approximately 2 cm incision; **c** Binostril approach: a part of bilateral posterior septal mucosa was necessarily resected. For one-and-a-half nostril and binostril approach, the assistant surgeon guided the endoscope through the right nostril while the senior surgeon manipulated the instruments through both nostrils

**Fig. 3:**
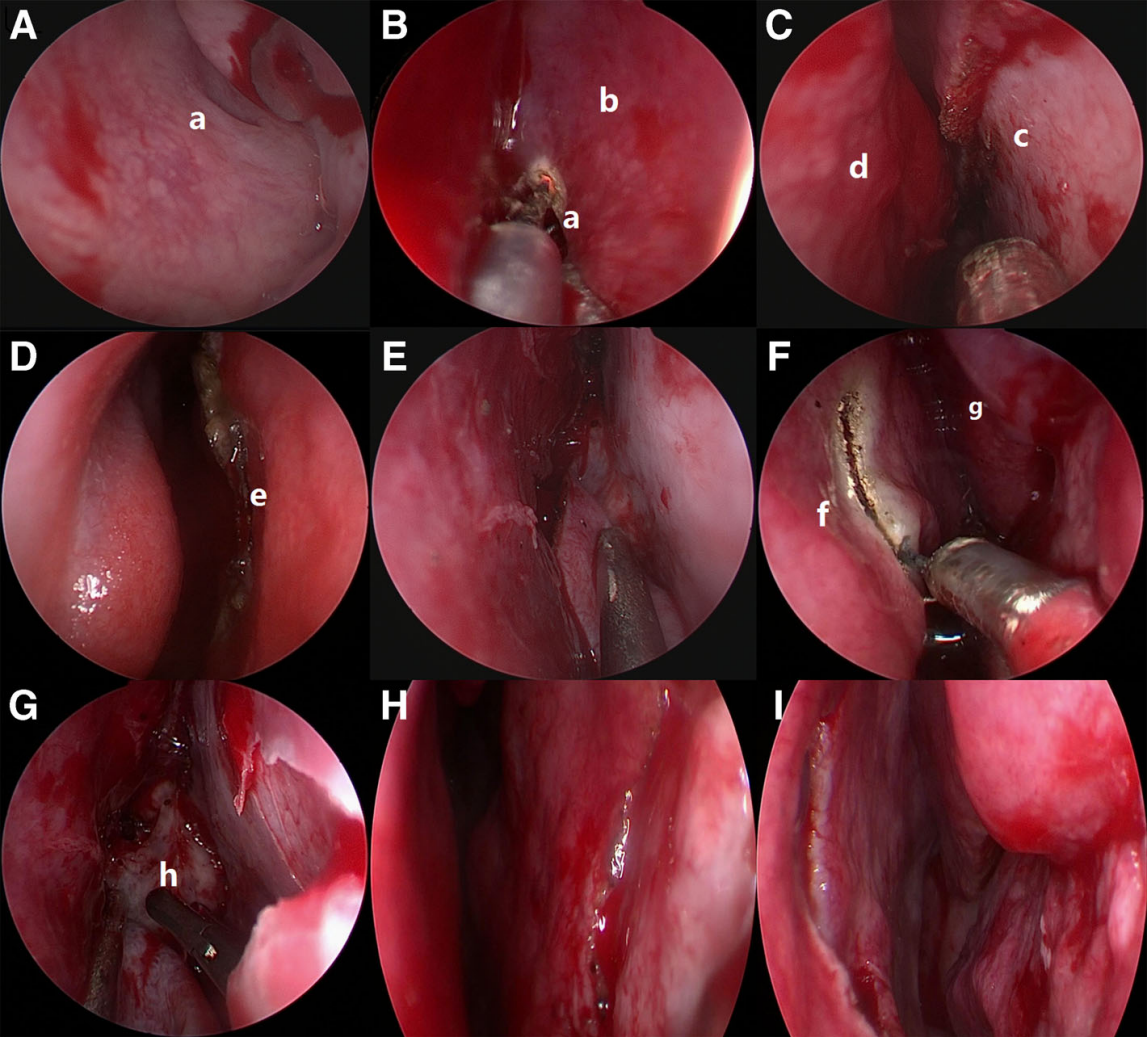
Intraoperative endoscopic images. **a** Localisation of the sphenoid ostium at the sphenoethmoidalrecessus. **b**–**d** Creating the right “rescue” nasoseptal flap- from 2 to 3 mm below the sphenoid ostium to the intercutaneomucous point of the nasal vestibule. **e** The nasoseptal flap was gently folded in the floor of nasal cavity. **f** A vertical incision of the left nasal septal mucosa was made. **g** A sufficient binasal access for two-surgeons using the four-handed technique. **h**–**i** The binasal mucosa and turbinates were placed back in normal anatomic position. a = sphenoid sinus ostium; b = medial nasal septum; c = the bony nasal septum; d = the right middle nansal turbinate; e = the intercutaneomucous point of the nasal vestibule; f = the vertical incision of the left nasal septal mucosa; g = the left middle nansal turbinate; h = the sphenoid rostrum

The last step was to incise the left nasal septal mucosa (Fig. 1b). The endoscope was introduced into the left nostril, and the left inferior and middle turbinates were out-fractured bilaterally. A vertical incision of the left nasal septal mucosa was made with unipolar electrocautery, which extended approximately 2 cm at the anterior-level of the middle turbinate (Fig. 3f). These procedures provided a sufficient binasal access for two surgeons using the four-handed technique (Fig. 3g). At the tumor resecting and sellar floor reconstruction stage, the assistant surgeon guided the endoscope through the right nostril while the senior surgeon manipulated the instruments through both nostrils (Fig. 2c).

For most pituitary tumors, the unilateral “rescue” nasoseptal flap was not necessary. The binasal mucosa and turbinates were placed back in normal anatomic position (Fig. 3h, i). If intraoperative cerebrospinal fluid (CSF) leakage had occurred, the unilateral “rescue” nasoseptal flap was then fashioned to ensure a vascularized repair. Nasal packing was performed for 24 h to prevent postoperative mucosal adhesion.

### The difference between META, BETA and OETA

The main difference was that the three approaches followed different methods for resecting the nasal septum mucosa. For the mononostril approach, a part of the unilateral posterior septal mucosa was need incised, the contralateral mucosa was integrally retained. All surgical instruments were manipulated only in one nasal cavity (Fig. 2a). For the binostril approach, a part of bilateral posterior septal mucosa was necessarily resected (Fig. 2b). For the one-and-a-half nostril approach, the unilateral septal mucosa required incising from 2 to 3 mm below the sphenoid ostium to the intercutaneomucous point of the nasal vestibule, and the contralateral mucosa only needed approximately 2 cm incision (Fig. 2c). Because the contralateral septal olfactory strip and the vascular pedicles of sphenopalatine were completely preserved, the risk of epistaxis and anosmia theoretically were decreased. For the one-and-a-half nostril and binostril approach, the assistant surgeon guided the endoscope through the right nostril while the senior surgeon manipulated the instruments through both nostrils.

## Results

### Patient demographics and tumor characteristics

Fifty-seven consecutive patients were included for analysis (35 men, 22 women). The mean age was 49.7 years (SD, 14.1 years; range, 18–83 years). The primary presenting symptoms were abnormal visual functions (56%), including vision loss and/or field-of-vision defect, followed by endocrinopathy (32%) and headache (23%). Twelve percent of adenomas were initially detected by cranial imaging for other symptoms, and eleven percent presented with pituitary apoplexy.

The mean volumes of the tumor were 22 × 20 × 20 mm^3^, and seven cases were recurrent. According to the Knosp scale [16], most tumors presented as Knosp grade 3 (33.3%), followed by Knosp grade 2 (28.1%) and grade 4 (19.3%). The fewest were grade 0 (5.3%) and grade 1 (14.0%). In 39 patients (68.4%), the tumor was non-secreting. The most prevalent secreting tumor was the producer of GH, in 12 patients (21.1%), followed by prolactinoma, in four patients (7.0%), and adrenocorticotropic hormone (ACTH), in two patients (3.5%). This data is in Table 1.

**Table 1:** Patient demographics and tumor characteristics

Patient age, mean ± SD, y	49.7 ± 14.1
Male/female, n	35/22
Follow up (range), mo	18 (6–24)
Tumor with volumes mm3	22*20*20
Recurrent, n (%)	7 (12.3%)
Presenting symptoms, n (%)	
Abnormal visual functions	32 (56%)
Endocrinopathy	18 (32%)
Headache	13 (23%)
Incidental	7 (12%)
Pituitary apoplexy	6 (11%)
Knosp score, n (%)	
0	3 (5.3%)
1	8 (14.0%)
2	16 (28.1%)
3	19 (33.3%)
4	11 (19.3%)
Nonfunctioning adenoma, n (%)	39 (68.4%)
Functioning adenoma, n (%)	18 (31.6%)
Growth hormone	12 (21.1%)
Prolactinomas	4 (7.0%)
ACTH	2 (3.5%)

### Surgical outcomes

Gross tumor resection (GTR) was determined by the absence of residual tumor on postoperative MRI. Postoperative imaging showed total gross removal in 45 (79%) patients. The gross total resection of patients was achieved in 100% of tumors of Knosp grade 0 to 2, 63.6% of grade 3, and 27.3% of grade 4.

Postoperative hormone evaluation showed that remission was achieved in 14 (77.8%) patients with secreting adenomas. Remission was achieved in 10 (83.3%) patients with growth hormone adenomas, in 3 (75%) patients with prolactinomas, and in 1 (50%) patient with ACTH adenomas. Postoperative neuro-ophthalmological evaluation revealed that 23 (72%) patients with previous vision loss and/or field vision defect were improved, with 9 (28%) unchanged (Table 2).

**Table 2:** Outcomes of surgery

	Gross Total Resection, n (%)
Overall	45 (79%)
Knosp score	
0	3 (100%)
1	8 (100%)
2	16 (100%)
3	7 (63.6%)
4	3 (27.3%)
	Hormonal remission rate, n (%)
Overall	14 (77.8%)
Growth hormone	10 (83.3%)
Prolactinomas	3 (75%)
ACTH	1 (50%)
	Visual outcomes, n (%)
Improved	23 (72%)
Stable	9 (28%)
Worse	0

### Complications

Complications are listed in Table 3. An intra-operative CSF leak was noted in 10 cases (17.5%), and all cases were reconstructed with a unilateral “rescue” nasoseptal flap. Two of these continued to leak post-operatively and were recovered 1 week after lumbar drainage. The seller dura of two cases were seriously injured during operation, but not broken. These two cases were also later reconstructed with a unilateral “rescue” nasoseptal flap. No postoperative CSF leak occurred in these two cases.

**Table 3:** Complications

Intraoperative cerebrospinal fluid leak	10 (17.5%)
The use of unilateral “rescue” nasoseptal flap	12 (21.1%)
Postoperative cerebrospinal fluid leak	2 (3.5%)
Temporary diabetes insipidus	3 (5.3%)
Anterior pituitary insufficiency	3 (5.3%)
Epistaxis	0

Other postoperative complications included temporary diabetes insipidus in three cases (5.3%), and anterior pituitary insufficiency in three cases (5.3%). There were no instances of postoperative arterial epistaxis or severe complications in the total cohort of 57 patients (Table 3).

### Postoperative follow-up

Seven cases were lost during follow-up and the mean follow-up time was 18 months (range 6 to 24). To investigate patients’ sinonasal complaints and health status, we conducted two questionnaires (Tables 4 and 5).

**Table 4:** The main sinonasal complaints’ questionnaire

Question 1: What are your main sinonasal complaints?	
Question 2: How do you feel about your sinonasal complaints?	
A. Worse than preoperation	B. Like preoperation

**Table 5:** The health status questionnaire

How do you feel about your health status?	
A. Worse than preoperation	B. Like preoperation

The main sinonasal complaints 2 weeks after surgery were: sense of smell damage (28%), sense of taste damage (4%), trouble breathing during the day (18%), thick nasal discharge (36%), post nasal discharge (8%), dried nasal material (6%), and headache (6%). Three months after surgery, there was no loss of sense of taste, postnasal discharge, and dried nasal material reported. Other complaints were decreased evidently: loss of sense of taste reduced to 4%, the rate of trouble breathing during the day decreased to 18%, the rate of thick nasal discharge decreased to 36%, and the rate of headache decreased to 2%. Six months after surgery, the main sinonasal complaints had nearly all returned to preoperative levels or better. One 72 years old patient did complain of trouble breathing during the day. The reason he complained of trouble breathing was nasal synechia. Through a simple operation of severing the nasal adhesion under endoscope, his nasal trouble was cured by an ENT doctor 9 months after surgery.

For health status, no patients reported being fully healed within 2 weeks after surgery. Fifteen patients (30%) recovered to preoperative status 3 months after surgery, and 48 (96%) patients recovered to preoperative status 6 months after surgery. Two patients with Knosp grade 4 tumors had radiation therapy after their surgery, and they recovered to normal status after 1 year postoperation (Table 6).

**Table 6:** Sinonasal quality of life and health status (follow up = 50)

Sinonasal complaints	Two weeks after surgery	Three months after surgery	Six months after surgery
Sense of smell damage	14 (28%)	2 (4%)	0
Sense of taste damage	2 (4%)	0	0
Trouble breathing day	9 (18%)	1 (2%)	1 (2%)
Thick nasal discharge	18 (36%)	5 (10%)	0
Post nasal discharge	4 (8%)	0	0
Dried nasal material	3 (6%)	0	0
Headache	3 (6%)	1 (2%)	1 (2%)
No sinonasal discomfort	1 (2%)	41 (82%)	48 (96%)
Health status			
Recovering to preoperative status	0	15 (30%)	48 (96%)

**Fig. 4:**
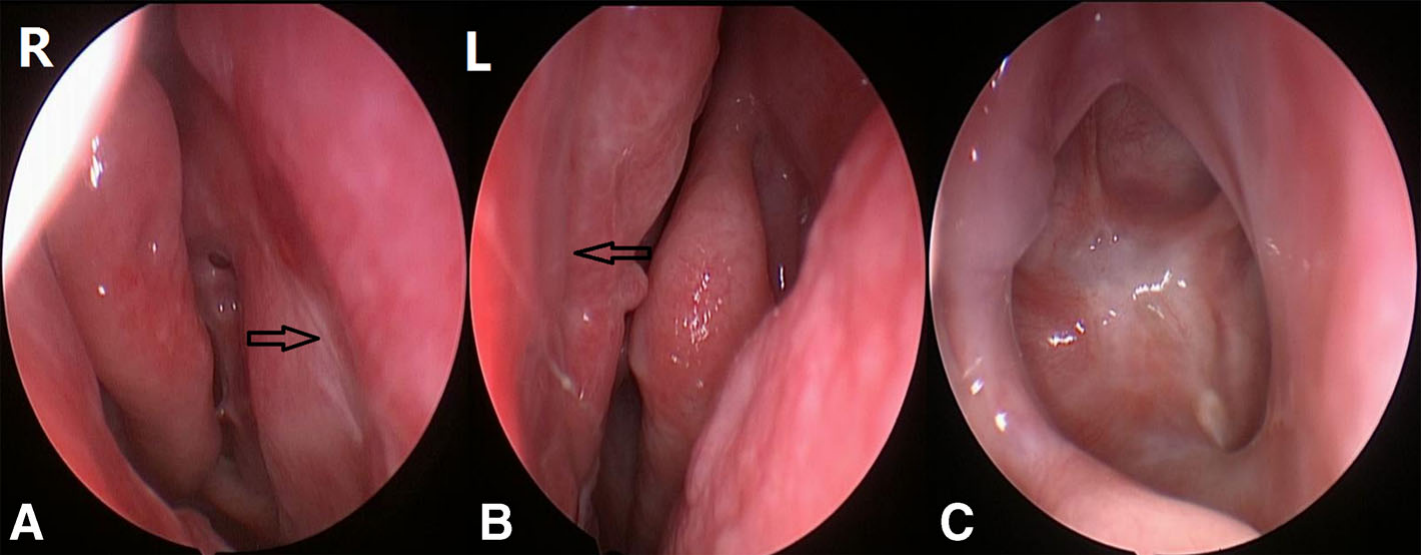
Postoperative outpatient follow-up after 3 months after surgery demonstrating the nasal mucosa favorable recovery and well prognosis. **a** the right nasal cavity, the *arrow* shows the right “rescue” nasoseptal flap interface. **b** the left nasal cavity, the *arrow* shows the interface of incision of the left nasal septal mucosa nasal. **c** The bottom of the saddle mucosa healed completely

Postoperative endoscopic visualization examination of nasal cavity was performed to collect information on 20 outpatients 3 months after surgery. The results demonstrated that the nasal mucosa was favorable to recovery and a positive prognosis (Fig. 4).

## Discussion

The two most commonly used endoscopic approaches for pituitary adenomas are the binostril and mononostril transsphenoidal approaches. Surgical outcomes, maneuverability of instruments, postoperative morbidity, and postoperative quality of life are the four most important principles that evaluate the efficiency of these two approaches. For general pituitary adenomas, some recent literature reported that the two approaches had similar results in terms of gross tumor resection rate, hormonal remission rate, improvement in visual function, CSF leak, and inception of diabetes insipidus [17, 18]. However, the binostril approach is superior in terms of surgical freedom and the resecting of invasive pituitary macroadenomas (such as tumors with parasellar and suprasellar expansion, and tumors requiring extended approaches) [19, 20]. There is additional damage to the nasal cavity that accompanies the binostril approach. Sinonasal complications, early postoperative sinonasal quality of life, and olfactory changes present significant negative impacts to the patient’s satisfaction with binostril transsphenoidal surgery. The methods limiting the sinonasal trauma of binostril approach are always of significance within our clinical work. We have created a one-and-a-half nostril endoscopic transsphenoidal approach, and this approach has proved highly effective through practice.

### The advantages of one-and-a-half nostril approach

#### Minimal invasiveness of nose: comparing OETA with BETA

In our opinions, OETA had less invasiveness to the nose, with the following advantages. First, BETA needed resecting of a part of the bilateral posterior septal mucosa, while OETA preserved a mostly intact left nasal septal mucosa. In the surgical procedure, the left nasal septal mucosa was only dissected approximately 2 cm. This vertical incision provided sufficient bi-nasal access and significantly reduced the invasion of the nasal septal mucosa. Second, for OETA, the right “rescue” nasoseptal flap preserved both the vascular pedicles of sphenopalatine or posterior nasal artery, as well as the septal olfactory strip. We made efforts to preserve the unilateral “rescue” nasoseptal flap. If intraoperative cerebrospinal fluid leakage had not occurred, the preserved unilateral “rescue” nasoseptal flap was successfully placed back in the normal anatomic position. Thus, theoretically, the risk of epistaxis and anosmia were also significantly decreased. For BETA, a part of the bilateral posterior septal mucosa was displaced, with the possibility of injury to the vascular pedicles of sphenopalatine and the septal olfactory strip. As a consequence, BETA may increase the risk of epistaxis and anosmia. In our series, there was no epistaxis, and no permanent anosmia. In 96 % of the patients the sense of smell returned to a normal level 3 months after operation. Moreover, 6 months after surgery, the main sinonasal complaints were nearly completely resolved. When comparing the epistaxis and permanent anosmia rates in our OETA series to the literature-reported BETA series, the former group seems to achieve better preservation of the normal anatomy and physiology of the nasal cavity.

In our series, in 96% of the patients the sense of smell returned to a normal level 3 months after operation. Moreover, 6 months after surgery, the main complaints of sinonasal quality of life were nearly completely resolved. In theory, for BETA, due to needing more extensive coagulation of the mucosa, the postoperative nasal cavity might require more time to restore to sound health. In the future, we will conduct a prospective case-control study to compare the sinonasal quality of life and health status between OETA and BETA.

### Maneuverability of instruments: comparing OETA with META

Our surgical outcomes were equivalent to or superior to those reported in the literature for endoscope based series [1, 3, 4, 7–9], including 79% gross tumor resection rate, 77.8% hormonal remission rate, and 72% improvement in visual function. In our opinion, this outcome may be attributable to the wide panoramic view of the endoscope, independent of both the width and depth of the access, which is not affected by the OETA. Importantly, compared to META, the OETA technique enhances the maneuverability of instruments because surgeons can obtain sufficient operating space via manipulation in both nasal cavities. Thus, with the assistant surgeon holding the endoscope, the primary surgeon can comfortably operate two instruments for bleeding control, tumor removal, and the reconstruction of the sellar floor.

For META, because of the space limitation in one crowded nasal corridor, the surgical freedom may be more affected by the presence of the endoscope and conflicts between the endoscope and dissecting instruments can arise. Especially, for macroadenomas with obvious parasellar invasion (Knosp grade 3 and 4), META has difficulty providing a direct view of the ipsilateral surgical target area using the ipsilateral nostril; it was a more favorable direction for the instruments coming from the opposite nostril. For OETA, changing the endoscope to the contralateral nostril could get the view of the ipsilateral surgical target area. In our series, even for Knosp grade 3 and 4 tumors, the rates of grossly complete resection were 63.6 and 27.3%, respectively (Table 7).

**Table 7:** Relevant parameters in this paper compared with those in the literature

Authors & Year	Cases	App	GTR		Complications			
			Knosp 0–2	Knosp 3–4	Csf	Tdi	Api	Ep
Cappabianca. P. et al. 2002 [7]	146	uni	62.3%		2%	5.5%	13.7%	1.4%
Rudnik, A. et al. 2007 [24]	70	uni	87.3%	6.7%	2.8%	5.4%	11.4%	1.4%
Zhang, Y. et al. 2008 [25]	678	uni	80.15		2.1%	/	/	0.3%
Bodhinayake, I. et al. 2014 [26]	64	uni	68.7%		4.1%	3.1%	4.7%	/
Bokhari, A. R. et al. 2013 [27]	79	bi	63%		3%	10%	/	/
López Arbolay, O. et al. 2013 [14]	278	bi	92.4%		1.4%	3.9%	3.6%	/
Paluzzi, A. et al. 2014 [28]	555	bi	67.1%		5%	/	2.9%	1.1%
Dallapiazza, RF.et al. 2015 [3]	80	bi	93.5%	28%	2.5%	/	/	1.3%
Present study	57	oeta	100%	33.3%	3.5%	5.3%	5.3%	0

*App* approach, *GTR* gross total resection, *Csf* cerebrospinal fluid leak; *Tdi* Temporary diabetes insipidus, *Api* Anterior pituitary insufficiency, *Ep* Epistaxis, *uni* unilateral, *bi* bilateral, *oeta* one and a half endoscopic transsphenoidal approach

Therefore, with satisfactory visualization, sufficient operating space, and superior maneuverability of instruments, the OETA achieved excellent outcomes for resection of almost all pituitary adenomas.

### The advantage of the unilateral “rescue” nasoseptal flap

With respect to postoperative morbidity, our results are comparable to that of other published series [1, 3, 4, 7–9, 20] in regards to factors such as postoperative CSF leakage (3.5%), anterior pituitary insufficiency (5.3%), and temporary diabetes insipidus (5.3%). In this study, the cases with the pituitary adenomas with Knosp grade 3 to 4 accounted for 52.6% of the total cases (30/57). In the surgical procedure of these cases, we observed 10 cases with tearing of the arachnoid membrane, which led to intraoperative CSF leak. We performed sellar packing with the unilateral “rescue” nasoseptal flap for the reconstruction in these cases. Just two of these cases developed into postoperative transient CSF leakage. The two cases were effectively treated with spinal CSF drainage for several days. These results show that the unilateral “rescue” nasoseptal flap had contributed to effective reduction of the incidence of postoperative CSF leak.

Many neurosurgeons have used bilateral nasoseptal “rescue” flaps as reconstructive materials [21–23]. Although this technique was reliable and efficient, the bilateral nasoseptal flap increased the degree of damage to nasoseptal mucosa. In our clinical experience, and in accordance with our results, the unilateral “rescue” nasoseptal flap was enough for most reconstruction. This will reduce the degree of damage to nasoseptal mucosa, as well as preserve the contralateral nasoseptal mucosa for the next potential re-operation.

## Conclusions

The one-and-a-half nostril endoscopic transsphenoidal approach for pituitary adenomas is a simple, yet reliable technique. It provides not only a sufficient surgical corridor for a 2-surgeon/4- or 3-hands technique, but also minimal invasion of the nose. Its clinical results with respect to surgical outcome, maneuverability of instruments, postoperative morbidity, and postoperative quality of life support the theory that this is a highly efficient technique.

## Abbreviations

BETA: Binostril endoscopic transsphenoidal approach
ETSS: The endonasal endoscopic transsphenoidal approach
META: Mononostril endoscopic transsphenoidal approach
OETA: One-and-a-half nostril endoscopic transsphenoidal approach
